# Intestinal obstruction in a mentally retarded patient due to pica

**DOI:** 10.1186/s12991-015-0060-4

**Published:** 2015-07-31

**Authors:** Hiroyuki Tokue, Yoichiro Takahashi, Satoshi Hirasawa, Sachiko Awata, Susumu Kobayashi, Takehiro Shimada, Azusa Tokue, Rie Sano, Yoshihiko Kominato, Yoshito Tsushima

**Affiliations:** Department of Diagnostic and Interventional Radiology, Gunma University Hospital, 3-39-22 Showa-machi, Maebashi, Gunma 371-8511 Japan; Department of Legal Medicine, Gunma University Hospital, Maebashi, Gunma Japan

**Keywords:** Intestinal obstruction, Mental retardation, Pica, Autopsy imaging, Postmortem computed tomography

## Abstract

A 40-year-old mentally retarded Japanese man was admitted at rehabilitation facility for handicapped persons and found dead in his bed. His neonatal period was complicated by seizures, and he had a medical history of schizophrenia. A postmortem computed tomography scan suggested an intestinal obstruction, but the cause was unknown. To clarify the cause of death, a medicolegal autopsy was carried out. The gastrointestinal tract was found to contain copious amounts of cloth pieces. A diagnosis of intestinal obstruction secondary to pica of clothes was made. Despite still being an essentially neglect condition; mental retardation is cause to significant burden to the patient, his relatives and caregivers and the whole society. Moreover, people with mental retardation may be at increased risk for potentially self-injury due to ingestion of non-eating substance or incongruent intake of eating substances, which may on turn lead to severe or even life-threatening medical and surgical complications as herein reported. Specific attention also to pica in mentally-retarded patients with sudden, severe, gastrointestinal events, should therefore be placed in order to prevent potential death or otherwise severe chronic consequences, ideally aiming at enhancing the early recognition and multi-disciplinary management of those psychological stressors or triggers potentially responsible for pica too.

## Background

Pica is the craving or ingestion of non-food items. The cravings found in patients diagnosed with pica may be associated with a nutritional deficiency state such asiron-deficiency anemia, with pregnancy, or with mental retardation or mental illness [1]. Pica is an eating disorder that occurs in both children and adults. Although pica is seen in the normal population, it is observed more frequently in individuals with mental retardation [2].

We present a case of bowel obstruction secondary to pica. The implication and need for an early multi-disciplinary approach are discussed in the present case-report. In addition, we reviewed previous reports of pica associated with mental retardation in adults. Pica should be suspected in mentally retarded patients with bowel obstruction of an unknown cause.

## Case presentation

**Fig. 1:**
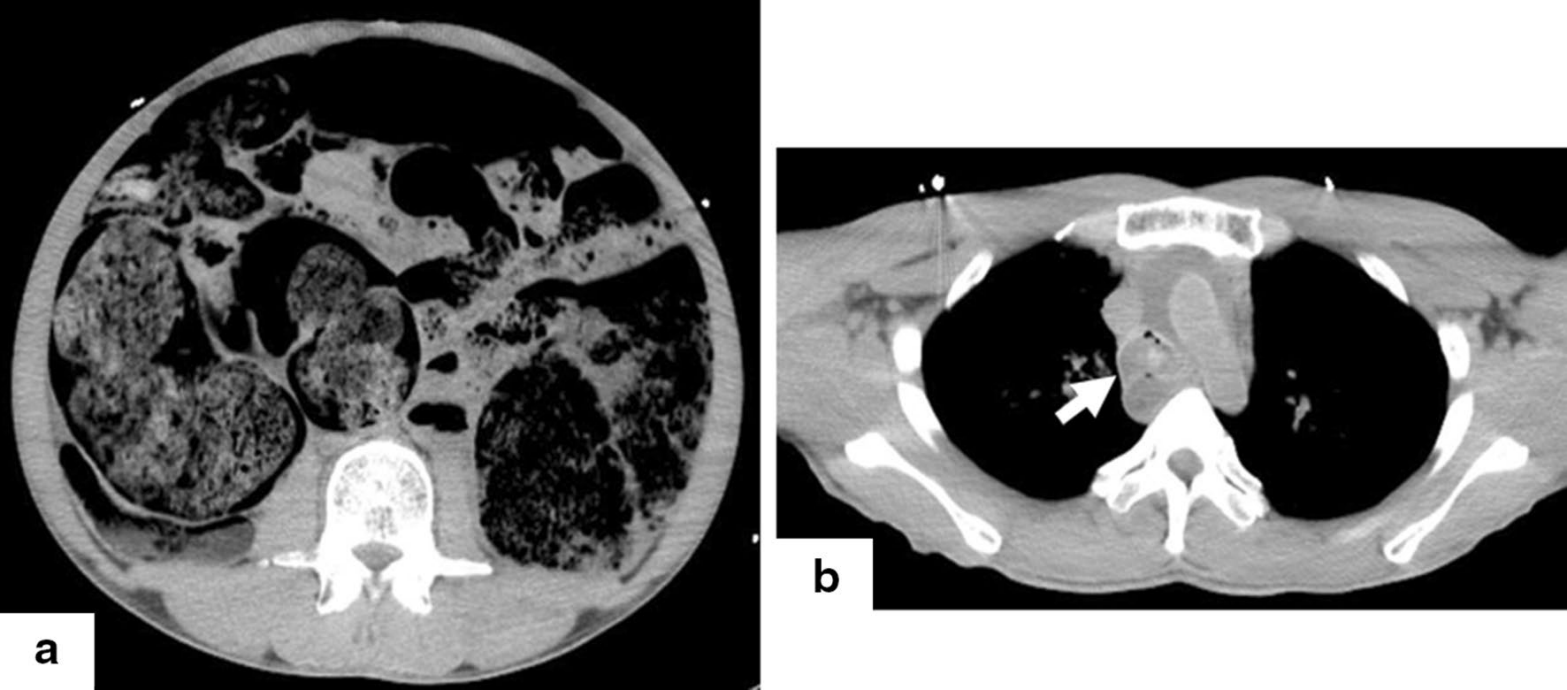
Postmortem computed tomography (PMCT) of a 40-year-old man with mental retardation. **a** Abdominal CT showing a distended small intestine and colon with an intraluminal heterogenous mass. **b** The trachea was filled with residue (*arrow*).

**Fig. 2:**
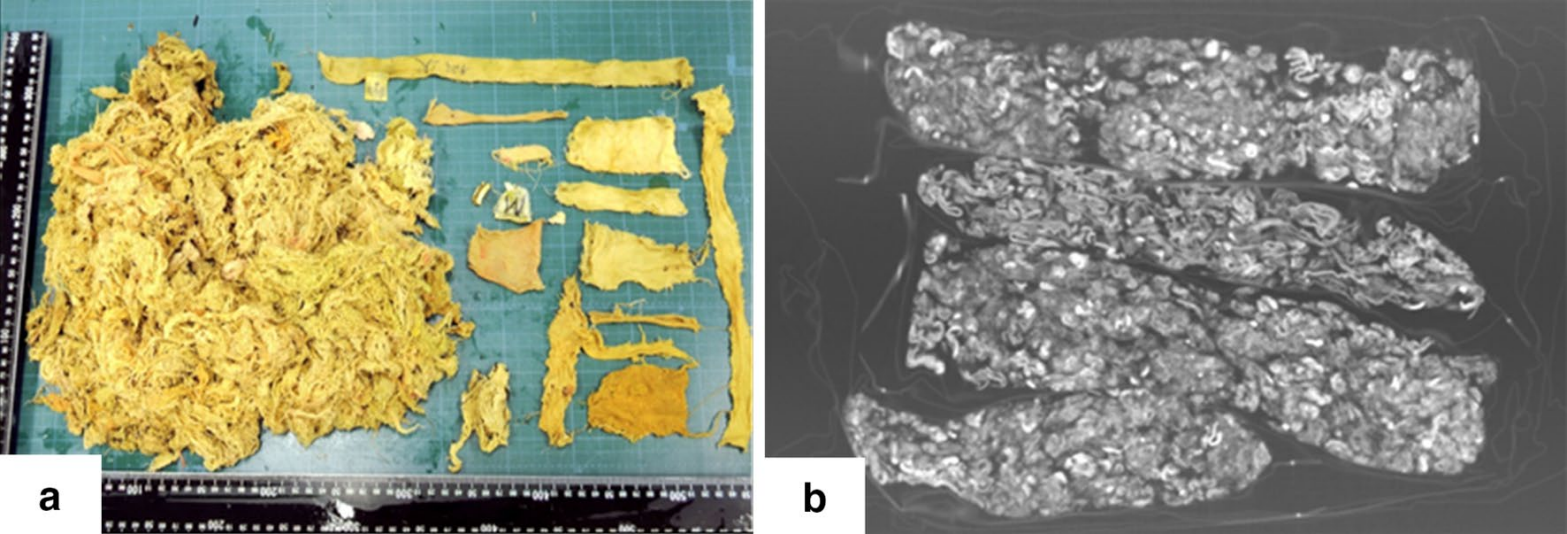
Image of gastrointestinal tract materials. **a** The gastrointestinal tract was found to contain copious amounts of cloth pieces (aggregate amount: 738 g). **b** A piece of cloth located in the intestinal tract was scanned by using computed tomography. However, this was not similar to the imaging findings of postmortem computed tomography.

A 40-year-old mentally retarded Japanese man was admitted at rehabilitation facility for handicapped persons and found dead in his bed. His neonatal period was complicated by seizures, and he had a medical history of schizophrenia. Intelligence quotient (IQ) level was 60. He spent in facility more than 20 years. Because the etiology of his death was unknown, a postmortem computed tomography (PMCT) scan was performed. The PMCT showed a massively dilated loop of bowel with stasis of fecal material in the small intestine and colon. Moreover, the trachea was filled with residue (Fig. 1). The PMCT of the patient determined the most plausible cause of the death as an intestinal obstruction and upper airway obstruction, but the cause was unknown. To clarify the cause of death, a medico-legal autopsy was carried out. The gastrointestinal tract was found to contain copious amounts of cloth pieces. There was also significant food residue in the patient’s respiratory tract. No other underlying organic diseases that could have caused or contributed to death were present. There was no evidence of trauma. We speculated that the internal pressure of the intestinal tract increased and induced a bowel obstruction due to pica, and that the patient died from an airway obstruction caused by mis-swallowing (Fig. 2).

## Discussion

Pica is the compulsive consumption of nonfood and/or non-staple food, often resulting in iron deficiency anemia [2]. It is important the pica is not just associated to mental retardation of a well-defined psychiatric diagnosis. It is more common in specific populations such as pregnant women, children aged 1–6 years, and people with developmental disabilities [3]. Pica is the most frequently observed eating disorder in people with developmental disabilities [2].

The cause of pica is unknown, but a multi-factorial etiology has been suggested. Causes include iron deficiency, psychological factors such as poverty, maternal neglect and abuse, lack of parental supervision, or a disorganized family situation, mental retardation, autism, and brain behavior disorders [4]. The various non-food forms of pica include amylophagia (laundry starch, corn starch), geophagia (clay, sand, dirt), lithophagia (stones, gravel, pebbles), pagophagia (ice), trichophagia (hair), and coprophagia (feces) [5].

Pica can result in intestinal obstruction. Anderson et al. reported a case of intestinal obstruction caused by talcum powder pica. Their literature review identified 43 previously reported cases of surgical complications caused by various forms of pica. Intestinal obstruction was the most common clinical presentation and the ileum was the site most commonly reported at surgery. Perforation with peritonitis was the next most common presentation. Clues pointing to pica as the underlying cause of abdominal complaints should not be overlooked, especially in patients who are known to be at a higher risk for pica [6].

Though potentially leading to severe medical complications or even life-threatening outcomes, relatively little evidence has been made available on the matter to date, especially among people with mental retardation, essentially due to stigma issues and lack of inclusion of people with mental disabilities in most controlled psychiatric and general medical trials. Therefore, the aim of the present case report is to provide a synthetic report of one of such cases in order to shed further light on the matter.

The prevalence of pica is difficult to establish because of differences in definition and the reluctance of patients to admit to abnormal cravings and ingestion. Various prevalence estimates have been reported in the literature. We reviewed the prevalence of pica associated with mental retardation in adults (Table 1), which showed variation across reports. Pica often goes unrecognized either because it is not included in history taking or because patients deny it. Danford et al. reported that the charts of 991 adults institutionalized for mental retardation included only 37 cases, whereas a survey conducted in the same population revealed that 256 (26%) of them had practiced pica [2]. In our case, the patient’s medical history did not include pica, and it was diagnosed only at autopsy.

**Table 1:** Summary data on prevalence of pica with mental retardation

Author	Pica definition	Method	Population setting (N)	Prevalence (%)
Matson et al. [7]	DSM-IV criteria	Psychological and functional assessment	Institution (790)	5.7
Applegate et al. [8]	Not stated, but not cooccurring with SIB, stereotypy, aggression, or ruminatio	Not mention	Developmental Center (417)	7.2
McAlpine et al. [9]	Eating non-food items and inappropriate food items	direct observation and/or review of medical problems	Institution (607)	9.2
Tewari et al. [10]	Eating non-food items and inappropriate food items	Information solicited from senior nursing staff	Learning Disability Hospital (246)	10.1
Swift et al. [11]	Frequent consumption of non-food and food related substances	Survey questionnaire, verbal questioning, and/or review of medical problems	Residential Facility (689)	22.1
Danford et al. [2]	Frequent consumption of non-food and food related substances	Staff interviews and/or direct observation	Institution (991)	25.8
Lohiya et al. [12]	Not mention	Not mention	Institution (323; 66)	77 (in 1977) 16.7 (in 1994)

*SIB* sever impairment battery, *DSM* diagnostic and statistical manual of mental disorders.

There are some cases of pica leading to death via intestinal obstruction. However, PMCT imaging findings of pica have not been reported. In recent years, computed tomography (CT) has proven to be an excellent diagnostic tool for the assessment of intestinal obstruction. It can accurately reveal the site and cause of obstruction, and, most importantly, distinguish between an extrinsic and an intraluminal cause. However, the diagnosis of pica is often difficult, even more so if the pica material is of low radiopacity. In our case, the obstructing material was cloth, and it was not recognized on CT. To confirm reproducibility, a piece of cloth located in the intestinal tract was scanned by using CT. However, this was not similar to the imaging findings of PMCT.

Pica is often observed in mental retardation. Pica should be suspected in mentally retarded patients with bowel obstruction of an unknown cause. If pica is suspected in the mentally retarded or in institutionalized patients, autopsy examinations should carefully check for the potential side effects of foreign material ingestion, which can range from upper airway obstruction to intestinal obstruction.

Despite still being an essentially neglect condition; mental retardation is cause to significant burden to the patient, his relatives and caregivers and the whole society. Moreover, people with mental retardation may be at increased risk for potentially self-injury due to ingestion of non-eating substance or incongruent intake of eating substances, which may on turn lead to severe or even life-threatening medical and surgical complications as herein reported. Specific attention also to pica in mentally-retarded patients with sudden, severe, gastrointestinal (G.I.) events, should therefore be placed in order to prevent potential death or otherwise severe chronic consequences, ideally aiming at enhancing the early recognition and multi-disciplinary management of those psychological stressors or triggers potentially responsible for pica too.

## Consent

Written informed consent was obtained from the patient’s legal guardians for publication of this case report and any accompanying images. A case report is not required institutional review board (IRB) in our institution.
